# Microglia and Monocytes/Macrophages Polarization Reveal Novel Therapeutic Mechanism against Stroke

**DOI:** 10.3390/ijms18102135

**Published:** 2017-10-13

**Authors:** Masato Kanazawa, Itaru Ninomiya, Masahiro Hatakeyama, Tetsuya Takahashi, Takayoshi Shimohata

**Affiliations:** 1 Department of Neurology, Brain Research Institute, Niigata University, Niigata 951-8585, Japan; ininomiya@bri.niigata-u.ac.jp (I.N.); hatakeyama.mas@gmail.com (M.H.); 2 Department of Neurology, Niishi-Niigata Chuo Hospital, Niigata 950-2085, Japan; ttetsuya.neurology@gmail.com; 3 Department of Neurology and Geriatrics, Gifu University Graduate School of Medicine, Gifu 501-1193, Japan

**Keywords:** stroke, microglia, monocyte, macrophage, pleiotropic effects, protective, polarization, M2-like

## Abstract

Stroke is a leading cause of morbidity and mortality worldwide, and consists of two types, ischemic and hemorrhagic. Currently, there is no effective treatment to increase the survival rate or improve the quality of life after ischemic and hemorrhagic stroke in the subacute to chronic phases. Therefore, it is necessary to establish therapeutic strategies to facilitate functional recovery in patients with stroke during both phases. Cell-based therapies, using microglia and monocytes/macrophages preconditioned by optimal stimuli and/or any therapies targeting these cells, might be an ideal therapeutic strategy for managing stroke. Microglia and monocytes/macrophages polarize to the classic pro-inflammatory type (M1-like) or alternative protective type (M2-like) by optimal condition. Cell-based therapies using M2-like microglia and monocytes/macrophages might be protective therapeutic strategies against stroke for three reasons. First, M2-like microglia and monocytes/monocytes secrete protective remodeling factors, thus prompting neuronal network recovery via tissue (including neuronal) and vascular remodeling. Second, these cells could migrate to the injured hemisphere through the blood–brain barrier or choroid–plexus. Third, these cells could mitigate the extent of inflammation-induced injuries by suitable timing of therapeutic intervention. Although future translational studies are required, M2-like microglia and monocytes/macrophages therapies are attractive for managing stroke based on their protective functions.

## 1. Introduction

Stroke is a leading cause of morbidity and mortality worldwide, and is categorized into two types, ischemic and hemorrhagic. The majority (70–80%) of stroke cases is ischemic, while intracerebral hemorrhage accounts only for 10–20% of all stroke cases [1]. Recently, several therapeutic strategies, such as thrombolytic treatments for acute ischemic stroke, were identified [2]. However, the patients who are eligible for the treatments are still between 3.4% and 5.2% of all patients with acute ischemic stroke because of the very narrow therapeutic time window [3]. To date, there is still no effective treatment that increases the survival rate or improves the quality of life after ischemic and hemorrhagic stroke [4,5,6] in the subacute to chronic phases. Currently, physical rehabilitation is considered the only effective therapeutic option to prompt functional recovery after stroke [7]. Therefore, it is necessary to establish other therapeutic strategies to facilitate the functional recovery in patients with stroke in the subacute and chronic phases.

“Single-target” therapies may be insufficient because ischemic and hemorrhagic cerebral injury involves several mechanisms. It has been proposed that therapeutic approaches should target multiple cell types to promote protection and recovery [8]. Cell-based therapies using bone marrow mononuclear cells or bone marrow-derived mesenchymal stem/stromal cells may be an effective “multi-target” therapeutic strategy to facilitate functional recovery in patients with stroke during the subacute and chronic phases through pleiotropic mechanisms [8,9]. One such mechanism observed in cell-based therapies, using neural progenitor cells and mesenchymal stem cells, was the induction of axonal outgrowth and angiogenesis through the secretion of vascular endothelial growth factor (VEGF) or brain-derived neurotrophic factor (BDNF) [10,11,12,13]. In addition, although it is not known whether the recovery environment modulated by cell-based therapies is a direct effect of the administered/transplanted cells themselves, peripheral immune cells may also alter the cytokines released from the periphery into the brain parenchyma [14]. For example, human umbilical cord-blood cell therapy might induce alternations, increasing the production of anti-inflammatory interleukin-10 (IL-10), and decreasing the production of pro-inflammatory interferon-γ (INF-γ) from splenocytes [15]. Although cell-based therapies against stroke are attractive, there remain several clinical concerns, including the distinct therapeutic mechanism and efficiency with which bone marrow-derived cells cross the blood–brain barrier (BBB) [16].

Cell-based therapies using microglia and monocytes/macrophages, and any strategies targeting these cells, might be a promising therapeutic strategy because these cells are the main source of the above-mentioned growth factors in the central nervous system (CNS) [8,17]. Although several studies have demonstrated that microglia and monocytes/macrophages might deteriorate the outcome after stroke in the acute phase [4,18,19,20], these cells are known to play protective roles through tissue and vascular remodeling after stroke, during both the subacute and chronic phases [20,21,22]. The protective M2-like microglia and monocytes/macrophages exert their effect through the secretion of remodeling factors, such as VEGF, BDNF, and matrix metalloproteinase-9 (MMP-9) [23,24,25], in addition to protective cytokines, such as transforming growth factor-β (TGF-β) and IL-10 [26,27], which may facilitate anti-inflammation, axonal outgrowth, and angiogenesis after ischemic [20,21,27,28] and hemorrhagic stroke [22,29,30]. Furthermore, microglia and infiltrating macrophages constitute the predominant phagocytes removing dead cells and tissue debris for remodeling after stroke [31]. In addition, administrated microglia and monocytes/macrophages can cross the BBB, particularly in the pathological condition [32,33,34]. Therefore, cell-based therapies using microglia and monocytes/macrophages preconditioned by optimal condition and/or any therapies targeting these cells might be an ideal therapeutic strategy for stroke.

In this review, we describe the pathophysiological roles of microglia and monocytes/macrophages after stroke. In addition, we briefly outline the therapeutic mechanisms of using microglia and monocytes/macrophages polarization for prompting neuronal network and angiogenesis, attenuating inflammation, and improving the therapeutic outcomes of patients with stroke.

## 2. The Differences and Similarities between Microglia and Monocytes/Macrophages

Microglia and monocytes/macrophages have distinct cellular origins. Microglia originate from yolk sac progenitors in the neuroepithelium, and express, in the adult brain, high levels of CX3C chemokine receptor 1 (CX3CR1), cluster of differentiation 11b (CD11b), and F4/80, low levels of CD45, and no C-C chemokine receptor type 2 (CCR2) [35]. In contrast, monocytes/macrophages originate from hematopoietic stem cells. The inflammatory monocytes/macrophages express CCR2, CD11b, Ly6C, and low levels of CX3CR1 [36]. After stroke, the release of nucleotides (ATP, UTP) from injured cells, including neurons, activates purinergic receptors on microglia and monocytes/macrophages, and leads to the production of pro-inflammatory cytokines [17]. Recently, the purinergic receptor P2Y12 (P2Y12R) has been identified as a specific marker for microglia in rodents, thus enabling their distinction from monocytes/macrophages [37]. Thus, the expression of P2Y12R might be specific for microglia, including human microglia [38,39]. However, both microglia and monocytes/macrophages express the same extent of cell surface markers (e.g., CD11b, F4/80, Iba-1) and share similar functions [40], including fundamental functions, such as phagocytosis and modulation of inflammation.

## 3. Microglia and Monocytes/Macrophages Act as Double-Edged Sword after Stroke

The function of microglia and monocytes/macrophages changes according to the different polarization states. Previously, several studies have demonstrated that microglia and monocytes/macrophages might play harmful roles in the acute phase of ischemic [18,19,20,41] and hemorrhagic stroke [42]. This was evidenced by in vitro experiments using microglial cultures stimulated with pro-inflammatory factors, such as lipopolysaccharide (LPS), IFN-γ, or tumor necrosis factor (TNF)-α, which demonstrated the death of cultured neurons in the presence of the activated microglia (or the resulting supernatant) [43,44]. Therefore, microglia and monocytes/macrophages were thought to accelerate inflammation. However, they have also been demonstrated to exert a protective role in processes involved in neurological recovery, including neurogenesis, axonal outgrowth, synaptogenesis, angiogenesis, oligodendrogenesis, and remyelination in several CNS diseases [45,46]. Based on these findings, microglia and monocytes/macrophages act as a double-edged sword, by playing essential roles in clearing debris, inflammation, causing tissue damage, and promoting tissue healing (remodeling) [22,27,47,48].

## 4. Phenotypic Polarization of Microglia and Monocytes/Macrophages after Stroke

**Figure 1:**
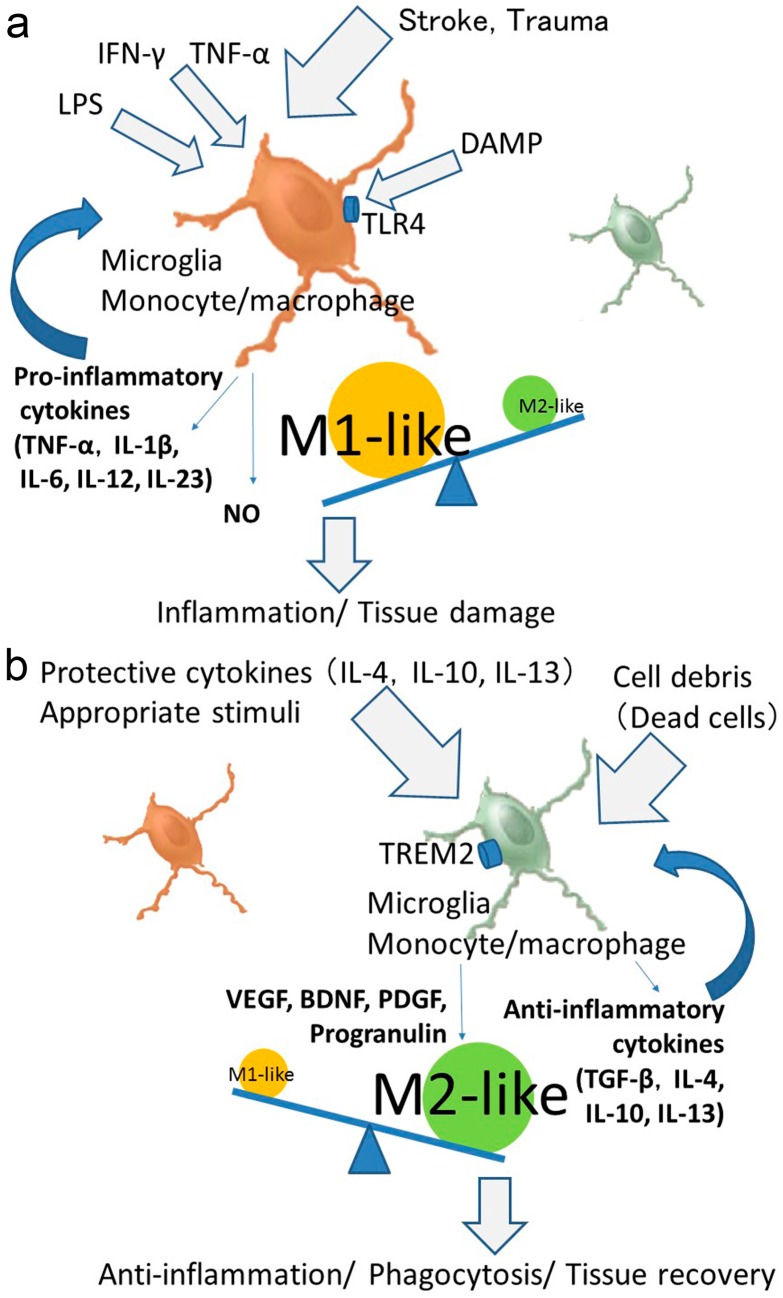
Scheme of M1-like or M2-like polarization. The M1-like responses as shown by the upregulation of pro-inflammatory cytokines such as interleukin (IL)-1β, IL-6, tumor necrosis factor (TNF)-α, and inducible nitric oxide synthase (iNOS). The M2-like responses as shown by the upregulation of markers such as arginase-1, chitinase-like protein 3 (also known as Ym1), cluster of differentiation (CD)206, CD163 and cytokines, IL-4, IL-10, transforming growth factor (TGF)-β, and growth factors. (**a**) Microglia and monocytes/macrophages polarize the M1-like state (classic, pro-inflammatory) following stroke, trauma, stimulation of lipopolysaccharide (LPS), interferon (IFN)-γ, TNF-α, or damage-associated molecular pattern (DAMP) through Toll-like receptor 4 (TLR4). The M1-like microglia and monocytes/macrophages would exacerbate inflammation and tissue damage. (**b**) Microglia and monocytes/macrophages polarize M2-like state (alternative, anti-inflammatory, protective) by protective cytokines and appropriate stimuli such as mild ischemia and drugs. Activation of triggering receptors expressed on myeloid cells 2 (TREM2) stimulates the phagocytic activity. Cell debris (dead cells) also stimulate microglia to polarize into the M2-like state. The M2-like microglia and monocytes/macrophages would suppress inflammation and prompt phagocytosis and tissue recovery. Abbreviations: BDNF, brain-derived neurotrophic factor; PDGF, platelet-derived growth factor; TGF-β, transforming growth factor-β; VEGF, vascular endothelial growth factor.

The activated microglia and monocytes/macrophages have been defined as either classic (pro-inflammatory; M1-like) or alternative (anti-inflammatory or protective; M2-like) under pathophysiological conditions. More precisely, M1-like microglia secrete pro-inflammatory cytokines, such as TNF-α, IL-1β, IL-12, IL-23, and nitrogen monoxide (NO), and exacerbate inflammation and tissue injury. In contrast, M2-like microglia secrete anti-inflammatory cytokines, such as TGF-β, IL-4, IL-10, Il-13, and growth factors such as VEGF, BDNF, platelet-derived growth factor (PDGF), and progranulin, suppress inflammation, and promote tissue recovery [49,50,51,52,53] (Figure 1a,b). Critical for the regulation of the immune response, the initial M1-like response is typically followed by a secondary M2-like activation that is important for wound healing and suppression of inflammation. This ideal concept is based on findings suggesting that IL-4 induced inflammatory macrophages adopt an alternative activation phenotype and reduce pro-inflammatory cytokine secretion in vitro [54]. Similar to macrophages, microglia also polarize to M1- or M2-like phenotypes [20,21,27]. In response to interferons (IFNs) from helper T cells [55], LPS or damage-associated molecular pattern (DAMP) stimulation through Toll-like receptor 4 (TLR4) [56,57], or IL-4/IL-13 signaling, the microglia and monocytes/macrophages undergo M1- or M2-like activations. The activation of the triggering receptor expressed on myeloid cells 2 (TREM2) stimulates the phagocytic activity in microglia and downregulates the expression of TNF-α and inducible nitric oxide synthase (iNOS) [58]. Opposing effects were obtained by TREM2 overexpression, while TREM2 deficiency attenuated the phagocytic activities of microglia and exacerbated the ischemic damage in experimental stroke [31] (Figure 1b). Thus, TREM2 is an anti-inflammatory receptor that simultaneously promotes a phagocytic activity. These results indicated that TREM2 might be an important player in controlling microglial M1/M2-like phenotypes. Moreover, recent studies have shown that exposure of classically activated macrophages (M1-like) to apoptotic cells causes a switch towards an alternatively activated M2-like phenotype of activated phagocytosis (the so-called “dead cell clearance hypothesis”) [59,60]. This concept, which is based on the functional skewing of mononuclear phagocytes, and occurs in vivo under physiological and pathological conditions, is likely truer for macrophages than for microglia, in which mixed phenotypes have been observed [61].

## 5. Influence of Age and Sex on the Polarized Activation of Microglia and Monocytes/Macrophages

Age and sex have been reported to affect polarization. Aged microglia derived from aged mice exhibit increased basal expression of TNF-α, IL-1β, and IL-6 [62]. These results indicated that the M1-like polarization was accelerated by aging. Moreover, in female mouse brains, ischemia-induced microglial activation enhanced the production of IL-6 via estrogen receptor signaling [63]. This result showed that the M1-like or M2-like polarization was modulated by sex hormones. Based on these observations, it becomes imperative to evaluate the effect of age and sex on microglia and monocytes/macrophages.

## 6. Polarized Activation of Microglia and Monocytes/Macrophages for Providing Therapeutic Strategies

Microglia are highly plastic cells that may rapidly transit between different states. Indeed, microglia have been shown to express both M1- and M2-like markers at the same time [23,40,64]. In addition, microglia and monocytes/macrophages might be categorized into four main states, namely the (1) classically activated M1-microglia with cytotoxic properties, (2) M2a-microglia with an alternate activation, which is involved in repair and regeneration, (3) M2b-microglia with an immunoregulatory phenotype, and (4) M2c-microglia with an acquired-deactivating phenotype [65]. Generally, M1-like microglia predominate at the injury site at the end stage of disease, when the immunoresolution and repair process of M2-like microglia are dampened. Although the polarization of M1/M2-like microglia during amyotrophic lateral sclerosis (ALS) may induce motoneuron degeneration or neuroprotection [66], isolated microglia from mutant superoxide dismutase 1-ALS mice revealed a microglial subpopulation between M1-like and M2-like phenotypes by transcriptome analysis [67]. In brain tissues from patients with Alzheimer’s disease, cluster analysis revealed that the frontal cortex of early Alzheimer’s disease samples was polarized to either the M1 or M2a neuroinflammatory phenotype, but not the M2-like phenotype [68]. In addition, tumor-associated microglia and monocytes/macrophages are also divided to several subpopulations [35,69]. The terminology and concept of the M1- and M2-like microglia and monocytes/macrophages might be complicated and disagreeable [70,71], and they might thus be oversimplified. However, several studies using rodents demonstrated that M2-like microglia and monocytes/macrophages improved the disease outcomes after therapeutic interventions, as described below. Thus, the polarized activation (M1- or M2-like phenotype) is a simple and desirable approach for providing therapeutic strategies in vivo.

## 7. Dynamic Polarized Changes of Microglia and Monocytes/Macrophages after Stroke

**Figure 2:**
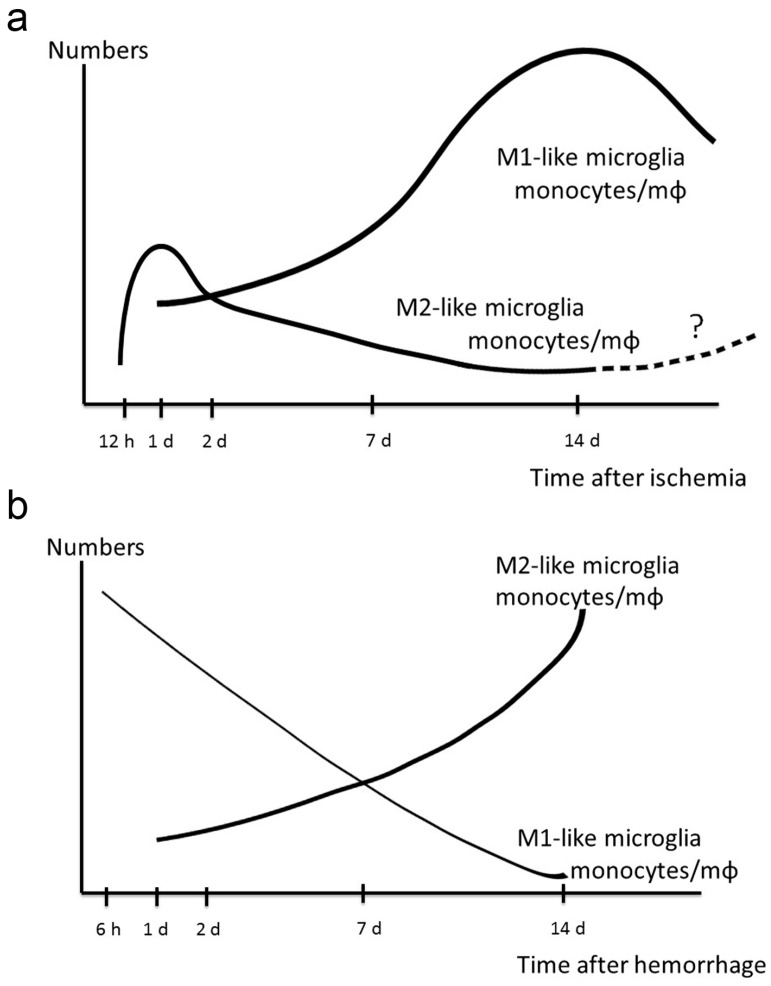
Dynamic polarized changes of microglia and monocytes/macrophages after stroke. (**a**) Temporal polarization changing by microglia and monocytes/macrophages after ischemic stroke by cell markers. The M1-like response exhibits an increasing trend in the first 14 days. The M2-like response exhibits a transient increasing trend in the first 1 to 2 days. Subsequently, the M2-like response exhibits a decreasing trend. However, it is unknown whether the M2-like response exhibits an increasing trend over the first 14 days. The balance of evidence supports an M2 to M1-like phenotype switch in the first 2 to 3 days. (**b**) Temporal polarization changing by microglia and monocytes/macrophages after hemorrhagic stroke. The M1-like response occurs as early as 6 h after hemorrhage, while the M2-like response starts to increase on day 1 after hemorrhage. Although a mixed M1- and M2-like microglial phenotype is evident during days 1 to 3, the balance of evidence supports an M1 to M2 phenotype switch in the first 7 days. The levels of most pro-inflammatory cytokines return to baseline on day 14. Abbreviations: mΦ, macrophage.

Resident microglia are major inflammatory cells in the brain, and are among the first cells to respond to brain injury [72,73]. Post-ischemic proliferation of microglia and monocytes/macrophages peaks at 48–72 h after focal cerebral ischemia and may last for several weeks after initial injury [19,21,53]. Temporal analyses of microglial phenotypes in ischemic animals demonstrated that M2-like microglia were detectable from 12 h, temporally increased at 1 to 3 days, and decreased several days after cerebral ischemia [20,21] (Figure 2a). On the other hand, M1-like microglia increased in the first 14 days after ischemic stroke.

Moreover, very little is known about the microglial and monocytic polarization after hemorrhagic stroke. Post-hemorrhagic temporal analysis of microglial phenotypes demonstrated that M1-like microglia and monocytes/macrophages increased acutely as early as 6 h after hemorrhagic stroke, and exhibited a decreasing trend in the first 14 days after hemorrhagic stroke [22,74]. In contrast, the microglial M2-like response increased on day 1 and exhibited an increasing trend in the first 14 days (Figure 2b). Although a mixed M1-like and M2-like microglial phenotype was evident on days 1 to 3, there is evidence that supports an M1- to M2-like phenotype switch during the first 7 days [30]. However, these results were obtained from animal models, and evidence of microglial and monocytes/macrophages polarization in human brains and blood samples after stroke is still lacking. Nevertheless, M1- to M2-like phenotype transition in the brain and blood may occur in patients after stroke. The results of the temporal change of cellular polarization demonstrated that increasing M2-like microglia and monocytes/macrophages during the acute phase would be an effective therapeutic strategy after both ischemic and hemorrhagic stroke.

## 8. Therapeutic Potential of M2-Like Microglia and Monocytes/Macrophages against Ischemic Stroke

**Figure 3:**
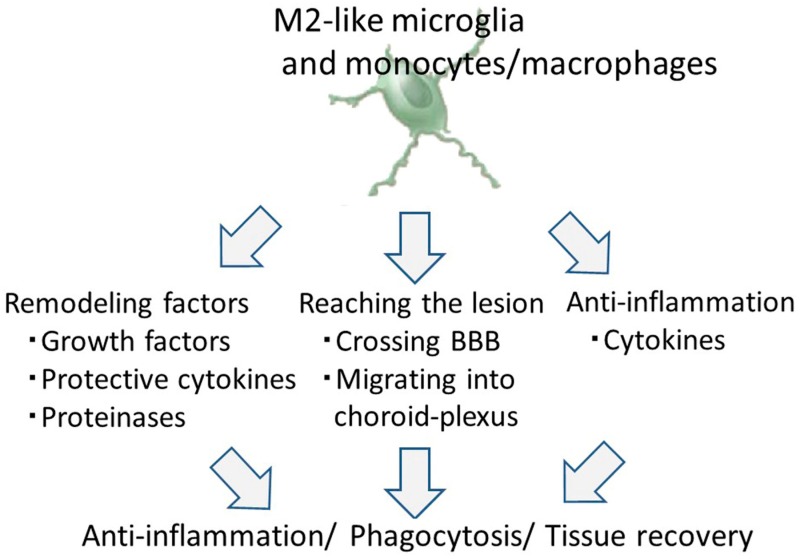
Illustration of the therapeutic effects of M2-like microglia and monocytes/macrophages. Abbreviations: BBB, blood-brain barrier.

Cell-based therapies using M2-like microglia and monocytes/macrophages might be protective therapeutic strategies against ischemic stroke based on three reasons (Figure 3).

### 8.1. Tissue and Vascular Remodeling by M2-Like Microglia and Monocytes/Macrophages

Because M2-like microglia and monocytes/macrophages secrete protective remodeling factors after ischemia, including VEGF, BDNF, progranulin, and TGF-β [10,24,28,53], they may facilitate axonal outgrowth and angiogenesis after ischemic stroke. There is direct evidence supporting a recovery-promoting effect of M2-like microglia and monocytes/macrophages following focal cerebral ischemia [28,75]. Indeed, M2-like microglia and monocytes/macrophages expressed VEGF, TGF-β, and insulin-like growth factor 1 (IGF-1) in the injury lesions, and were shown to enhance axonal growth of corticospinal motor neurons [28,76]. Additionally, non-primed, not M2-like microglia and monocytes/macrophages had no effect on the recovery of sensorimotor function at 4 weeks or 3 months post-insult [28,75]. These data indicated that the M2-like state of microglia and monocytes/macrophages is essential for the recovery promoting effect. This is in agreement with previous findings reported following the depletion of infiltrating monocytes/macrophages after ischemic stroke abolished long-term behavioral recovery and drastically decreased tissue expression of anti-inflammatory genes, including TGF-β and CD163 [34]. In addition, axonal outgrowth was observed to exert active effects on the surrounding angiogenesis. After the administration of M2-like microglia, remodeling factors such as VEGF, TGF-β, and MMP-9 are secreted in the brain parenchyma, thus resulting in angiogenesis [28]. It has been also demonstrated that metformin treatment may induce M2-like polarization and result in angiogenesis following stroke [77]. Therefore, the recovery-promoting effect is most likely due to secreted factors from M2-like microglia and monocytes/macrophages that can promote tissue remodeling (axonal outgrowth and angiogenesis).

Although the administration of M2-like monocytes/macrophages prompted functional recovery, the infarct volume was not reduced by the cell therapy [75]. The release of IL-10 by M2-like microglia enhanced dendritic spine formation and synaptogenesis in cultured neurons [78]. Furthermore, microglia have been reported to release TNF-α, which increased in turn, spine density [79]. Interestingly, microglial contact directly induced synapse formation in the developing cortex [80]. Thus, microglial P2Y12R might be necessary for synaptogenesis following brain injury [81]. These changes may be behind the M2-like microglia and monocytes/macrophages enhanced functional synaptogenesis in the remaining neurons. In normal adult brain, chondroitin sulfate proteoglycan, a component of the extracellular matrix, has been reported to inhibit axonal outgrowth [82], and is shown to be cleaved and degraded by MMP-9 [83]. Microglia is the main source of MMP-9 [84] and cathepsin L after ischemia [85]. These proteinases might degrade the extracellular matrix and prompt axonal outgrowth. Recently, it was reported that growth factor progranulin knockout microglia reduced synaptic formation [86]. We also reported microglial secretion of progranulin after ischemia [53]. These results support the notion that secretion of growth factors, cytokines, and proteinases by microglia and monocytes/macrophages directly enhances neuronal repair. Thus, M2-like microglia and monocytes/macrophages would prompt neuronal network recovery through tissue remodeling (including neuronal tissue) by growth factors, cytokines, and proteinases.

### 8.2. Infiltrating Properties of M2-Like Microglia and Monocytes/Macrophages

The ideal characteristics of cell-based therapies and any medication is the possibility of crossing the BBB and reaching the injured brain parenchyma, which is a feature of M2-like microglia and monocytes/macrophages. The accumulation of microglia and infiltration of monocytes/macrophages into the brain after ischemic stroke has been demonstrated by translocator protein (TSPO) as a biomarker of positron emission tomography (PET) [87], and ultrasmall superparamagnetic particles of iron oxide (USPIO) as a biomarker of magnetic resonance imaging (MRI) [88]. Pathophysiologically, the adhesion receptor macrophage-1 antigen (Mac-1) mediates the adhesion of microglia and monocytes/macrophages to the endothelial surface. This effect might be important for the infiltration of microglia and monocytes/macrophages into the affected brain parenchyma [89,90]. Moreover, the upregulation of Mac-1 following the preconditioning of M2-like microglia to oxygen-glucose deprivation enabled the microglia to cross the BBB and reach the injured brain parenchyma [28]. Several studies have demonstrated that the chemokine stromal-derived factor-1 (SDF-1, also known as CXCL12) plays a role in the homing of microglia and monocytes/macrophages, as well as stem cells, to the areas of ischemic injury [91,92,93]. In addition, M2-like monocytes/macrophages could infiltrate the ischemic hemisphere via the choroid plexus–cerebrospinal fluid route [75]. Interestingly, M2-like microglia and monocytes/macrophages, but not the non-primed or non-M2-like microglia and monocytes/macrophages, could migrate into the ischemic hemisphere through the BBB [28] or choroid–plexus [75]. This ability of the M2-like microglia and monocytes/macrophages is crucial for the success of cell-based therapies.

### 8.3. The Anti-Inflammation Effect of M2-Like Microglia and Monocytes/Macrophages

Both M2-like microglia and monocytes/macrophages can suppress inflammation. Neuroinflammation occurs several days after ischemic stroke and within 7 days after hemorrhagic stroke, which may be the consequence of increased M1-like microglia and monocytes/macrophages [18,19,20,21]. Administration of M2-like microglia and M2-like polarization by IL-4 may suppress inflammation and enhance functional recovery after ischemic stroke [28,94]. Therefore, M2-like microglia and monocytes/macrophages could suppress the extent of inflammation-induced injuries by therapeutic intervention. Thus, interventions by M2-like microglia and monocytes/macrophages are ideal approaches for suppressing inflammation.

## 9. Previous Reports on the Effects of M2-Like Microglia and Monocyte/Macrophage against Ischemic Stroke

Studies on direct administration of primary microglia, cell-line microglia, and monocytes/macrophages against ischemic stroke have been reported [24,28,34,95,96,97,98]. Although some reports demonstrated the suppression of neuronal cell death and/or improvement of functions [24,28,34,95,96,97], other studies reported no effect [98] (Table 1).

However, most previous evaluations did not take M1/M2-like cellular polarization into consideration, although we reported intravascular administration of M2-like microglia preconditioned by optimal oxygen-glucose deprivation directly improved functional outcome after ischemic stroke [28]. Additionally, subcutaneous [99] or intraventricular [94] administration of IL-4 induced the polarization of endogenous microglia and monocytes/macrophages to M2-like characteristics. The results improved functional outcomes after ischemic stroke. On the other hand, intravenous administration of IL-4 induced neutrophilic hyperresponse and allergic reaction [100]. Recently, it was reported that selective and unselective monocyte/macrophage depletion and M1- and M2-like macrophage transfer did not influence tissue damage and functional outcomes within 42 days after cerebral ischemia [101].

In addition, several clinical trials using mononuclear cells and stem cells similar to monocytes/macrophages, have been undertaken to test their safety and preliminary efficacy in patients with ischemic stroke (Table 2). According to the ClinicalTrials. gov trial registry (https://clinicaltrials.gov/), four trials were completed [102,103]. However, they did not use polarized cells. Additionally, other open trials using mononuclear cells and mesenchymal stem cells have been conducted. However, only one prior trial using blood mononuclear cells and M2-like macrophages has been conducted [102,103,104,105,106,107]. Further studies are warranted for therapeutic strategies using microglia and monocytes/macrophages against ischemic stroke, to evaluate the optimal timing of the interventions, compare microglia or monocytes/macrophages therapies, and determine how to stimulate cells and polarize their status.

## 10. Therapeutic Potential of M2-Like Microglia and Monocytes against Hemorrhagic Stroke

Microglia and monocytes/macrophages act as phagocytes to scavenge debris in the CNS. The M2-like microglia and monocytes/macrophages, which play an important role in hematoma clearance, healing, and neuroprotection, might be a reasonable candidate therapy for the recovery from hemorrhagic stroke [29,74]. The scavenger receptor CD36 contributes to the increasing phagocytic ability of microglia and monocytes/macrophages [108]. Furthermore, CD36 deficiency might lead to TLR4 upregulation and IL-10 secretion. The TLR4-mediated phagocytosis might contribute to microglial polarization (polarized to M1-like) and function. Although both M1-like and M2-like microglia and macrophages express phagocytic receptors, M2-like microglia and macrophages may present a stronger phagocytic ability to remove dead neurons than the M1-like phenotype [109,110]. On the other hand, the M1-like phenotypes may cause neuronal loss by increasing phagocytosis of viable neurons, because of producing high levels of reactive oxygen species (ROS) [49,111,112,113]. In contrast, the M2-like phenotypes trigger anti-oxidative responses after stroke by suppressing the levels of ROS. Moreover, M2-like phenotypes induced anti-oxidative responses by increasing the levels of glutathione-SH (GSH) and heme oxygenase-1 [114,115,116,117]. Thus, phagocytosis mediated by M2-like microglia and monocytes/macrophages may be neuroprotective, whereas that mediated by M1-like microglia and monocytes/macrophages may result in neuronal damage. However, whether modulating this polarization might resolve hematoma and improve functional recovery remains to be elucidated.

Currently, several medications are undergoing clinical trials (reviewed by Lan et al. [22]). These medications are reported to either prompt decreasing levels of M1-like microglia (e.g., fingolimod, deferoxamine) [118,119] or increasing levels of M2-like microglia (e.g., Chinese medicine sinomenine) [120]. However, cell therapies using microglia and monocytes/macrophages have yet to be investigated.

## 11. Conclusions

In a recent report, microglia-like cells have been derived from cultured human embryonic stem (ES) and induced pluripotent stem (iPS) cells, and a robust and efficient protocol for the rapid production of microglia-like cells was established [121]. Microglia/monocytes from iPS cells might be a potential candidate for therapeutic applications in stroke, provided that the tumorigenesis issue in cells derived from iPS cells is resolved. Cryopreservation negatively affects the cellular viability of cells. Both fresh and frozen monocytes/macrophages have similar beneficial effects in a rodent ischemic stroke [122]. The use of both autologous and allogenic cells raises the possibility that mononuclear cells (monocytes/macrophages) could potentially be stored as a banked source for possible therapeutic use when the need arises. Microglia and monocytes/macrophages are thought to potential cell therapies for several CNS diseases, psychiatric diseases [123], Alzheimer’s disease [124], and traumatic brain injury [125]. Although future translational studies are required, microglia and monocytes/macrophages therapies are attractive targets to manage stroke based upon their protective functions.

## Figures and Tables

**Table 1:** The list of cell therapies using microglia and monocytes/macrophages for ischemic stroke.

Reference	Source	Polarization	Stimuli	Comments
Kanazawa, et al. Sci Rep 2017 [28]	Primary microglia	M2-like microglia	OGD	Improving outcome by axonal outgrowth and angiogenesis Upregulation of VEGF, TGF-β and MMP-9
Wattananit, et al. J Neurosci 2016 [34]	Monocyte-derived macrophages	M2-like macrophage	None	Improving outcome
Womble, et al. Molecular Cell Neurosci 2014 [97]	Umbilical cord blood mononuclear cells	No polar	None	Reduced infarct volume and improving outcome
Desestret, et al. PLoS ONE 2013 [98]	Bone marrow-derived monocytes	M2-like macrophage	IL-4	Not reduced infarct volume and no improving outcome
Jiang, et al. Brain Res 2013 [96]	Bone marrow-derived mononuclear cells Primary microglia	No polar	None	Reduced infarct volume and improving outcome by mononuclear cells No improving outcome by microglia
Narantuya, et al. PLoS ONE 2010 [95]	Microglial cell line, HMO6	No polar	None	Reduced infarct volume and improving outcome
Imai, et al. JCBFM 2007 [24]	Primary microglia	No polar	None	Inhibition of neuronal cell death Upregulation of BDNF and GDNF

Abbreviations: BDNF, brain-derived neurotrophic factor; GDNF, glial cell-derived neurotrophic factor IL-4, interleukin-4; MMP-9, matrix metalloproteinase-9; OGD, oxygen-glucose deprivation; TGF-β, transforming growth factor-β; VEGF, vascular endothelial growth factor.

**Table 2:** The list of clinical trials using mononuclear cells for ischemic stroke.

Reference	ClinicalTrials.gov Identifier	Source	Polarization	Stimuli	Comments
Prasad, et al. Stroke 2016 [102]	NCT01501773	Autologous bone marrow stem cell	No polar	None	No beneficial effect
Sharma, et al. Stroke Res Treat 2014 [103]	NCT02065778	Autologous bone marrow mononuclear cell	No polar	None	Improving outcome
	NCT00950521	Autologous peripheral blood stem cell (CD34+)	No polar	None	Not reported results
	NCT00473057	Autologous bone marrow cell	No polar	None	Not reported results
Chernykh, et al. Cell Transplant 2016 [104]	-	Autologous blood mononuclear cell	M2-like macrophage	GM-CSF	Improving outcome
Taguchi, et al. Stem Cell Dev 2015 [105]	-	Autologous bone marrow mononuclear cell	No polar	None	Improving outcome
Friedrich, et al. Cell Transplant 2012 [106]	-	Autologous blood mononuclear cell	No polar	None	Improving outcome
Honmou, et al. Brain 2011 [107]	-	Autologous mesenchymal stem cell	No polar	None	Improving outcome

Abbreviations: GM-CSF, granulocyte macrophage colony-stimulating factor.

## Abbreviations

ALS	amyotrophic lateral sclerosis
BBB	blood–brain barrier
BDNF	brain-derived neurotrophic factor
CCR2	C-C chemokine receptor type 2
CD	cluster of differentiation
CNS	central nervous system
CX3CR1	CX3C chemokine receptor 1
DAMP	damage-associated molecular pattern
ES	embryonic stem
GDNF	glial cell-derived neurotrophic factor
GM-CSF	granulocyte macrophage colony-stimulating factor
GSH	glutathione-SH
IFN	interferon
IGF	insulin-like growth factor
IL	interleukin
iNOS	inducible nitric oxide synthase
iPS	induced pluripotent stem
LPS	lipopolysaccharide
Mac-1	macrophage-1 antigen
MMP-9	matrix metalloproteinase-9
MRI	magnetic resonance imaging
NO	nitrogen monoxide
OGD	oxygen-glucose deprivation
PDGF	platelet-derived growth factor
PET	positron emission tomography
P2Y12R	P2Y12 receptor
ROS	reactive oxygen species
SDF-1	stromal-derived factor-1
TGF-β	transforming growth factor-β
TLR4	Toll-like receptor 4
TNF-α	tumor necrosis factor-α
TREM2	triggering receptor expressed on myeloid cells 2
TSPO	translocator protein
USPIO	ultrasmall superparamagnetic particles of iron oxide
VEGF	vascular endothelial growth factor
